# Supplementary material for: Prevalence and factors associated with delayed antiretroviral therapy initiation among adults with HIV in Alebtong district, Northern Uganda: A facility-based study
Source: PLOS Glob Public Health. 2022 Aug 8;2(8):e0000691. doi: 10.1371/journal.pgph.0000691 (PMC10021445; doi:10.1371/journal.pgph.0000691)
Supplement: S1 Text — (PDF) [file pgph.0000691.s001.pdf]

Structured questionnaire

Title: Predictors for late antiretroviral therapy initiation among diagnosed HIV positive patients in Alebtong District, Uganda

Section A: Introduction

Sub-County…………………… Parish…………………………….

Village………………………….. Health facility name…………………

Name of interviewer………………………… Respondent's Code……………

Date of interview…………………………. Time of interview………………………….

Section B: Background Information

1. What is your age (in completed years)…………………….
2. Sex of respondent

   1 = male 2 = female
3. What is the level of your highest education?

   1= no education 2= primary education

   3= secondary education 4= post-secondary education
4. What is your occupation?

   1= peasant farmer 2= informal employment

   3= formal employment
5. Where is your area of residence?

   1= rural area 2= urban area
6. What is your current marital status?

   1= not married 2= married 3= widowed 4= separated
7. What is your religion?

   1= Catholic 2= Protestant 5= other, specify………………

   3= Muslim 4= Born again

Section C: Individual Factors

8. What was the reason for doing an HIV test?

   1= to know my HIV status 2= my spouse was diagnosed with HIV

   3= had developed signs and symptoms of HIV 4= others, specify………………………………

9. a) Did you feel depressed/frustrated with the outcome of your HIV result? (If no proceed to qn. 10.a)

   0 = no 1 = yes

   b) If yes, why? 1= didn't know I had it 2= Fear of dying soon 3= Fear of spouse

   4= Fear of stigma 5= Fear of ARV side effects 6= others

10. a) When you found that you were HIV positive, did you disclose your HIV status to anyone? (If no proceed to qn. 11.a)

   0= no 1= yes

   c) How long did it take you to disclose your HIV status?……………………..

   b) If yes, to whom did you first disclose your HIV status to?

   1= my spouse 2= my children

   3= my father 4= my mother

   5= my friend 6= others, specify……………………..

11. a) Did you go back to the facility on the date you were told to go back? (If ever missed tick no, if never missed tick yes), (If yes proceed to qn. 12.a)

   0= no 1= yes

   b) If no, why 1= was still depressed/frustrated 2= fear of people's opinion

   3= didn't want people to know 4= others, specify……………

12. a) Do you drink alcohol? (If no, proceed to qn. 13.a)

   0= no 1= yes

   b) If yes, how much in a week?

   1= daily 2= two times a week

   3= three times a week 4= four times a week

   5= five times a week 6= six times a week

13. a) Do you smoke tobacco? (If no, proceed to qn. 14.a)

   0= no 1= yes

   b) If yes, on average how many sticks do you smoke per day? ....

14. a) Do you think ARV treatment is beneficial to you?

   0= no 1= yes

   b) If yes, how?

   1= prolong life 2= prevent AIDS

   3= reduce viral load 4= others, specify………………………..

15. On average, what time does it take you to travel from your home to the place where you got the HIV test? (in minutes)…………………………..
16. a) How would you rate satisfaction with the services you received when you went for an HIV test?

    1= Never 2= Rarely 3= sometimes 4= most for the time 5= All the time

    b) If never, what is your main reason? (Give only one main reason)

    ………………………………………………………………………………………

Section D: Community factors

17. Does your household have the following properties? (circle all those mentioned)

    1= permanent building, yes/no 2= vehicle, yes/no

    3= electricity, yes/no 4= semi-permanent building, yes/no

    5= motorcycle, yes/no 6= television, yes/no

    7= animals, yes/no 8= bicycle, yes/no

    9= radio, yes/no

    Note: High socio-economic status (Includes all items from 1-3), medium socio-economic status (includes all items from 4-6) and low socio-economic status (includes all items from 7-9)

18. How far (in kilometres) is your home from the health facility? ……………....
19. a) Do you always use traditional medicine for treatment when you are sick? (If no, proceed to qn. 20)

    0= no 1= yes

    b) If yes, which one?

    1= herbal medicine 2= witch doctor's spirit

    3= spiritual healing/church 4= bone setters

    5= other, specify………………..
20. Does your culture believe in the use of ARV for treatment of HIV?

    0= no 1= yes 2= I don't know
21. a) Do you receive any support from the community as regards your sickness (HIV)? (If no, proceed to qn. 22.a)

    0= no 1= yes

    b) If yes, what support?

    1= material support 2= prayers

    3= encouragement/advice 4= transport to facility

5= other, specify………………………………

22. a) Have you ever experienced any form of violence from your spouse because of being HIV positive? (If never, proceed to qn. 23.a)

1= Never 2= Rarely 3= Sometimes 4= Most of the time 5= All the time

b) What form of violence? Physical Violence Y/N (Any form of violence constitutes physical violence)

1= Pushed, shook or throw something at her/him

2= Slapped her/him

3= Twisted her/his arm or pulled her/his hair

4= Punched her/him with his/her fist or with something that could hurt her/him

5= Kicked, dragged or beat her/him up

6= Tried to choke her/him or burn her/him on purpose

7= Threatened her/him or attacked her/him with a knife, gun or other weapon

c) What form of violence? Sexual Violence, Y/N (Any form of violence constitutes sexual violence)

1= physically forced her/him to have sexual intercourse when she/he did not want to

2= physically forced her/him to perform any other sexual acts she/he did not want to

3= forced her/him with threats or any other way to perform sexual acts she/he did not want to

d) What form of violence? Emotional Violence, Y/N (Any form of violence constitutes emotional violence)

1= Said or did something to humiliate her/him in front of others

2= Threatened to hurt or harm her/him or someone she/he cared about

3= Insulated her/him or made her/him feel bad about herself/himself

e) Experienced all the above 3 types of violence

0= No 1= Yes

23. a) Have you ever experienced any form of stigma when people heard that you were HIV positive?

1= Never 2= Rarely 3= Sometimes 4= Most of the time 5= All of the time

b) If yes, how?

1= some avoided to greet me 2= refusal to talk to me

3= refusal to share items with me 4= Rejection by relatives/friends

5= other, specify………………………….

24. a) After HIV test, did anyone make a follow-up to know your condition or remind you of the appointment to go back to the facility?

    0 = no 1= yes

    b) If yes in 35 a) above, who made the follow-up?

    1 = health workers 2 = VHT 3 = expert client

    4 = Linkage facilitator 5 = others, specify………………………………..

Section E: Health service factors

25. Did you take an HIV test from this health facility? (If yes, proceed to qn. 27)

    0 = no 1 = yes

26. If no, where did you take the HIV test from?

    1= government hospital 2 = government health centre

    3= private hospital 4= private clinic

    5= drug shop 6= PNFP (faith based) facility

    7= other, specify……………………..

27. Were you counselled before taking an HIV test?

    0= no 1 = yes

28. Were you counselled after taking an HIV test?

    0= no 1 = yes

29. What was the average waiting time (in minutes) for the HIV test? ....

30. a) Did you receive your HIV test result on the same day the test was taken? (If yes, proceed to qn. 31.a) 0= no 1= yes

    b) If no, after how long (in days) did you receive your HIV result?.......................

31. a) Were you given ARV drugs the same day you did the HIV test?

    0= no 1 = yes

    b) What was the reason for not giving you ARV drugs the same day you did HIV test?

    1 = Lack of ARVs 2 = was not counselled 3 = had other sickness (Malaria, TB, Hepatitis) 4 = Too weak to swallow drugs 5 = Not made my mind

    6= others, specify……………………………………

32. Were you given any in-kind support from the health facility because of your status (HIV+)?

    0= no 1= yes

    b) If yes in 32 (a) above, what in-kind support did you receive?

    1 = food 2 = sugar 3 = porridge

    4 = jerry-can 5 = water guard/tablet 6 = mosquito net

7 = others, specify…………………………………

33. Is there a waiting space/shade at the facility when waiting for ART services?

    0= no   1= yes

34. How would you rate the attitude of health workers towards patients who are HIV positive?

    1 = very poor   2 = poor   3 = fair

    4 = good   5 = very good

The End

Thank you for your time!
