# Supplementary material for: Prevalence and factors associated with delayed antiretroviral therapy initiation among adults with HIV in Alebtong district, Northern Uganda: A facility-based study
Source: PLOS Glob Public Health. 2022 Aug 8;2(8):e0000691. doi: 10.1371/journal.pgph.0000691 (PMC10021445; doi:10.1371/journal.pgph.0000691)
Supplement: S2 Text — (PDF) [file pgph.0000691.s002.pdf]

Original translation of structured questionnaire

Nying Ikweda: Apeny bot jo ame tye kede silim ame tye ajamo yat iye Alebtong Distrik

<u>Section A: Anyut</u>

Gombola…………………………… Janjago……………………………

Wangtic…………………………… Nying dakatal………………………….

Nying ngat apeny…………………. Numba agam apeny……………….

Ninodwe…………………. Cawa……………………..

<u>Section B: Ngec Okene</u>

1. Mwaka nit ye adi? ....
2. Ico onyo dako?
   1= ico 2= dako
3. Ikwano igik I kilaci adi?
   1= pe akwano 2= primary
   3= cinia 4= akato cinia
4. Itio ngo?
   1= abedo apur 2= atio ocara
   3= atio leja leja
5. Ibedo kwene?
   1= Icalo 2= itawun
6. Onyo inyomere?
   1= pe anyomere 2= anyomere
   3= opokere oko
7. Dini ni obedo mene?
   1= otoli 2= ogeri
   3= acilam 4= alare
   5= en okene…………………….

<u>Section C: Kop Ikom Dano Acel Acel</u>

8. Tyen kop ango omio i pimo remo pi silim?
   1= me ngeo ka atye kede silim 2= cwara/cega onwongo tye kede silim
   3= angut me two silim onwongo tye 4= en okene………………………
9. a) Onyo adwogi me pimere obin oturu cunyi?
   0= pe 1= oturu cunya
   b) Ka oturu cunyi, pingo?
   1= Pe onwongo angeo ni atye kede 2= lworo too con
   3= lworo cwara/cega 4= lworo pokere kede cwara/cega
   5= lworo yat silim 6= en okene……………………

10. a) ikare ame inwongo ni itye kede silim, ikobi ngatoro?
    0= pe 1= akobo
    b) Kai kobo, ikobi nga numba acel?
    1= cwara/dakona 2= otino na
    3= papa 4= tota
    5= wati na 6= owote na 7= en okene…………………………

11. a) Abin idok idakatal ikare ame okobi ni idok iye?
    0= pe 1=aee
    b) K ape, pingo?
    1= onwongo cunya otur atek 2= lworo lok ajo
    3= onwongo pe amito ni jo nge 4= en okene…………………………

12. a) Imato kongo?
    0= pe 1= amato
    b) Ka imato, imato aromene I cabit acel?
    1= didik 2= iryo cabit acel
    3= idek cabit acel 4= ingwen cabit acel
    5= ibic cabit acel 6= ibicel cabit acel

13. a) Imato taba?
    0= pe 1= amato
    b) Ka imato, imato gin adii inino acel?
    1= acel 2= aryo
    3= adek 4= angwen
    5= abic 6= akato abic

14. a) Itamo ni konyo ayat silim tye boti?
    0= pe 1= tye do
    b) Kat ye, tye ningo?
    1= medo kwo 2= gengo twoe okene
    3= dwoko wel kudi ping 4= en okene……………………

15. Iyeng kede kony ame omi ikare ame iwoto pimo silim?
    0= pe 1= ayeng kede

16. Itamo ni otero dakika adi me ya ituri tuno ika pimere me silim?
    1= pe tuno dakika 30 2= dakika 30-60
    3= dakika 61-90 4= dakika 91-120
    5= kato dakika 120

Section D: Kop Akwako Cominiti

17. Paco ni tye kede jami ni? (gur ducu en okobo)
    1= ot abati ocwe kede bricks, tye/pe 2= otoka, tye/pe
    3= eletrisity, tye/pe 4= ot ocwe kede lobo, tye/pe
    5= opik, tye/pe 6= televson, tye/pe
    7= leyi, tye/pe 8= gali, tye/pe
    9= radio, tye/pe

18. Ya ituri tuno I dakatal borere rom mene?
    1= pe tuno kilomita 5 2= kato kilomita 5

19. a) imaro tic kede yat me tekwaro ka komi lit?
0= pe 1= amaro tic kede
b) Ka imaro tic kede, mene?
1= yat me tekwaro 2= tipo ajoka
3= cuny acil ikanica 4= orwa jo
5= en okene…………………

20. Tekwaro ni ye me tic kede yat silim?
0= Pe 1= Ee

21. a) Inwongo konyoro I bot otedero akwako two silim?
0= Pe 1= Anwongo
b) Ka inwongo, kony ango?
1= jami 2= lego
3= odio cunya 4= gi wot I dakatal
5= en okene………………………….

22. a) Inwongo kit gero more me yi ot acalo adwogi me yin ibedo kede silim?
1= pe 2= Anonok 3= Cawa okene 4= Cawa apopol 5= Cawa ducu

b) Kit gero ango? Gero me apwod, Ee/Pe (Acel iye nwongo obedo gero me apwod)
1= Acor, lworo onyo obai kede ginoro 2= Abap
3= Omino cingi onyo ojako yer wi 4= Odongi onyo ojwati kede ginoro ager
5= Ogwei, owayi onyo opwodi 6= Otemo deyi onyo wangi kede mac
7= Oburi onyo ocubi kede pala, obai kede oduku onyo ginoro ager

c) Kit gero ango? Gero me mit? Ee/Pe (Acel iye nwongo obedo gero me mit)
1= Omaki teteko me codi ame pe imito 2= Omaki tetek me timo ginoro akwakere kede mit
3= Omaki tetek onyo otimo ginoro akwakere kede mit

d) Kit gero ango? Gero me tam? Ee/Pe (Acel iye nwongo obedo gero me tam)
1= Okobo onyo otimo ginoro me wango yi ikom lwak 2= Oburi ni iwani onyo wano ngat yin imaro
3= Oyeti onyo owango yei
e) Onyo inwongo gero adek ducu imalo nu? 0= Pe 1= Ee

23. a) Itamo ni jo obedo kede apoka poka ikomi ikare ame gin owinyo ni itye kede silim?
0= pe 1= jo obedo kede

b) Ka jo obedo kede, iyore ango?
1= jo okene onwongo lworo mota 2= jo okene kwero lok keda
3= jo okene kwero noto jami keda 4= owote na odoko anok
5= en okene………………………..

24. a) Iyonge pimere pi silim, onyo ngatoro abin owopo yori me ngeo kit itye kede onyo pe?
0= pe 1= Ee
b) Ka Ee, nga abin owopo yori me poyo wi?

1 = Dakatal 2 = Jo ame ocoyo ikan paco 3 = Jo ame two silim

4 = VHT 5 = Jo okene, kobi……………………………………………

Section E: Kop akwako dakatal

25. Ipimo remo me silim idakatal man?
    0 = Pe   1 = Ee

26. K ape ipimo silim kan, Ipimo silim ikwene?
    1= dakatal agamente   2= dakatal agamente atino
    3= dakatal ape mega agamente   4= klinik
    5= ka cato yen   6= dakatal a misson (PNFP)
    7= en okene…………………………..

27. Omado cunyi apwod peru opimo silim?
    0= Pe   1= Ee

28. Omado cunyi iyonge pimo silim?
    0= Pe   1= Ee

29. Itamo ni otero cawa adii ikuru pimere pi silim? ………………………
30. a) Abin omi adwogi me pimere iceng ipimere kede?
    0= pe   1= omia iceng a pimere kede
    b) Ka pe, omi awene I kara ame I pimere kede?
    1= iyi cabit acel   2= iyi cabit aryo
    3= iyi cabit adek   4= iyi cabit angwen
    5= iyonge cabit angwen

31. a) Abin dakatal omi yat me two silim inino ame ipimere kede?

    0= Pe   1 = Ee

    b) Itamo ni ngo omio pe omi yat silim iceng ame ipimere kede?

    1 = yat two silim onwongo otum oko   2 = onwongo pe omado cunya
    3 = onwongo atye kede two okene (two atipa, TB, Hepatitis B)   4 = onwongo koma lit atekateka
    5 = onwongo pwod pe amoko tama   6 = men okene, kobi………………
32. a) Abin omi konyoro idakatal akwako bedo kede two silim?

    0= Pe   1= Ee

    b) Ka Ee, kong ango ame omi?

    1 = cem me acama   2 = cukal   3 = nyuka
    4 = jerikan   5 = yat me neko kudi ipi   6 = tanaru me ober
    7 = men okene, kobi…………………………………
33. Onyo ka bedo aber ame oyubo pi weo otwo ka akuru kede kony tye?

    0= Pe   1= Tye

34. Itamo ni dakatale leo kop kedwu aber ka ibino wunu me nwongo kony?

    1 = ra atek   2 = rac   3 = ber
    4 = ber atek   5 = ber tutwal

Agiki

Apwoyo me gamo apeny!
