# Supplementary material for: Prevalence and factors associated with delayed antiretroviral therapy initiation among adults with HIV in Alebtong district, Northern Uganda: A facility-based study
Source: PLOS Glob Public Health. 2022 Aug 8;2(8):e0000691. doi: 10.1371/journal.pgph.0000691 (PMC10021445; doi:10.1371/journal.pgph.0000691)
Supplement: S1 Table — (PDF) [file pgph.0000691.s003.pdf]

Data Collection on Health Service Factors

Health facility name………………….. Title of respondent…………………………

Date of data collection………………… Time of data collection………………………

Questions	Response
1. a) Do the facility have current guideline on ART?	Yes/no
1. b) Is the guideline being followed at the ART clinic?	Yes/no
2. a) What is the recommended ART clinic days in a week according to the Ministry of Health, Uganda?	……………
2. b) How many clinic days do you have in a week?	……………
3. a) What record keeping arrangement for patients do you practice here?	1= arranged alphabetically<br>2= arranged according to geographical location<br>3= arranged according to sex<br>4= arranged according to period (monthly, quarterly, and yearly), 5= Randomly
3. b) Do you have cases of lost files (for patients) here in this ART clinic?	0= no 1= yes
4. a) Are there cases of ARV stock-outs in this facility?	0= no 1= yes
4. b) If yes, for how long was the facility without ARVs, say in the last 6 months?	…………………
4. c) What do you think are some of the causes of ARV stock-outs?	1= inadequate supply, 2= increased initiation of patients on ART, 3= misuse of ARVs, 4= delayed supply of ARVs, 5= expiry of ARVs, 6= others…………..
5. a) What is the required number of ART clinic staff according to Ministry of Health, Uganda?	……………………..
5. b) Current number of staff in the ART clinic?	…………………..
5. c) If no, why?	1= Inadequate staff, 2= few patients,<br>3= lack of recruitment, 4= transfer of staff, 5= staff turnover, 6= others…………………
6. a) Are there cases of work overload in this clinic?	0= no 1= yes
6. b) If yes, why?	1= inadequate staff, 2= too many patients, 3= absenteeism, 4= workshop, 5= on leave, 6= others……………
7. a) Are there cases of staff absenteeism in this clinic?	0= no 1= yes
7. b) If yes, why?	1= conflicts at work, 2= sick leave, 3= maternity leave, 4= annual leave, 5= workshop, 6= others……..
8. a) Do the clinic have enough space?	0= no 1= yes
8. b) Do the clinic have waiting space for patients?	0= no 1= yes
9. a) What is the order of attending to patients in this ART clinic?	1= first come first serve, 2= random, 3= according to sitting order, 4= those too ill patients are served first, 5= others………………
9. b) Do you think the patients are happy with the order of service ?	0= no 1= yes
