# Supplementary figures and images for: Prevalence and factors associated with delayed antiretroviral therapy initiation among adults with HIV in Alebtong district, Northern Uganda: A facility-based study
Source: PLOS Glob Public Health. 2022 Aug 8;2(8):e0000691. doi: 10.1371/journal.pgph.0000691 (PMC10021445; doi:10.1371/journal.pgph.0000691)

A flow chart showing sampling procedure

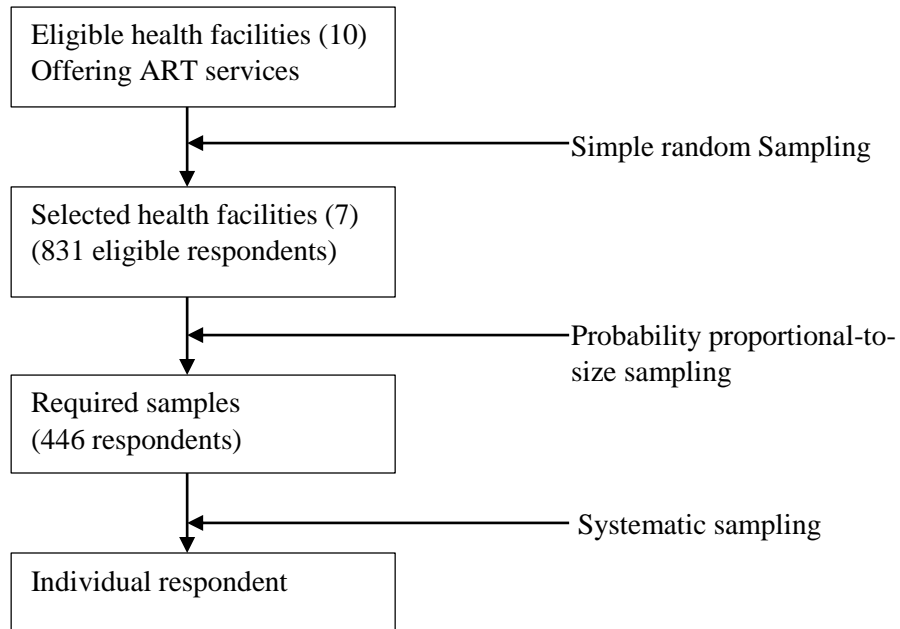

Supplement: S1 Fig — (PDF) [file pgph.0000691.s004.pdf]
