# Supplementary material for: Prevalence and factors associated with delayed antiretroviral therapy initiation among adults with HIV in Alebtong district, Northern Uganda: A facility-based study
Source: PLOS Glob Public Health. 2022 Aug 8;2(8):e0000691. doi: 10.1371/journal.pgph.0000691 (PMC10021445; doi:10.1371/journal.pgph.0000691)
Supplement: Attachment — Submitted filename: Response to Reviewers.docx [file pgph.0000691.s005.docx]

**Response to Reviewers**

**Journal Requirements**

1. Please include additional information regarding the survey or questionnaire used in the study and ensure that you have provided sufficient details that others could replicate the analyses. For instance, if you developed a questionnaire as part of this study and it is not under a copyright more restrictive than CC-BY, please include a copy, in both the original language and English, as Supporting Information.

**Response 1**

Additional information regarding the questionnaire has been provided in the methods section under data collection procedures and measurements. The questionnaire in both Original and English language version including data extraction tool are provided in the attachment as supporting information.

2. Please amend your Financial Disclosure statement. If you did not receive any funding for this study, please simply state: “The authors received no specific funding for this work.”

**Response 2**

The financial statement has been amended as indicated on the first page of the manuscript.

3. Please update your Competing Interests statement. If you have no competing interests to declare, please state: “The authors have declared that no competing interests exist.”

**Response 3**

The competing interest statement has been updated as specified on the first page of the manuscript.

4. Please provide a complete Data Availability Statement in the submission form. If your research concerns only data provided within your submission, please write “All data are in the manuscript and/or supporting information files.” as your Data Availability Statement.

**Response 4**

The research only concerns data provided within our submission and the data availability statement is provided on the first page of the manuscript.

5. We have noticed that you have a list of supporting information legends in your manuscript. However, there are no corresponding files uploaded to the submission. Please upload them as separate files with the file type 'Supporting Information'. Please ensure that all Supporting Information files are included correctly and that each one has a legend listed in the manuscript after the references list.

**Response 5**

Supporting information have been provided as separate files in the attachments.

**Responses to reviewer 1**
**Comment 1.** The authors should be using people first language. HIV-infected/HIV-positive/HIV patients are no longer acceptable terms to use in the literature due to their stigmatizing effects. Please use ‘person living with HIV’ or ‘person with HIV.’

**Response 1.** Authors have used the first language in writing the manuscript. We have replaced the terms HIV-infected/HIV-positive/HIV patients with either persons living with HIV or person with HIV throughout the text.

**Comment 2.** Abstract: inaccurate spell out of HIV abbreviation and no spell out of AIDS abbreviation in introduction. Should use updated stats if possible. The conclusion should not simply repeat the results but rather it should consider the future implications of the findings in the context of prior literature.

**Response 2.** The right spelling of HIV abbreviation has been provided in the text and a statement on AIDS included in the introduction section of the abstract.

**Comment 3.** The data availability statement needs revising. The data are not in the text. Did the authors mean to say that the instructions for accessing the data are included in the text?

**Response 3.** The data availability statement has been revised as shown on the first page of the manuscript.

**Comment 4.** Introduction – need to define delayed ART initiation sooner; what is the cost of ART in Uganda?

**Response 4.** Delayed ART initiation definition has been brought forward to the first paragraph of the introduction section. ART is provided for free in the public health facilities in Uganda, however, the cost is unknown in the private health facilities.

**Materials and Methods**
**Comment 5.** Study context: Prevalence estimates are inconsistent with the introduction.

**Response 5.** The HIV prevalence estimate in the introduction was a typo but has been corrected to 6.8% and moved under study context.

**Comment 6.** Line 252 do you mean ‘registries’ rather than ‘registers’?

**Response 6.** We meant registries and it has been corrected in the text.

**Comment 7.** Lines 282-288: excessive and unimportant info included about the choice of method. Recommend shortening.

**Response 7.** The portion has been shortened as recommended.

**Comment 8.** Line 295: states models 1, 3 and 4 have 7 demographic vars, but model 2 only has 6. What demographic variable was removed from model 2 and why?

**Response 8.** A total of 7 demographic variables were added to model 1 and remains 7 as indicated on table 4. However, 7 individual variables were added to model 2 but only 6 of them considered, one variable (tobacco smoking) was removed from the model due to the multicollinearity effect, i.e. had a variance inflation factor of more than 10 value. The value of less than 10 VIF was the cut-off for our consideration.

**Comment 9.** The rationale for trying each of these models is needed. Why block 1 always is included but block 4 variables are only tested in the final model? What about the other possible combinations? Including those that break apart the “blocks” into smaller components? Did you consider a variable selection procedure that maximizes model performance while penalizing for the number of variables included? (e.g. AIC or BIC).

**Response 9.** In hierarchical modeling, each block/model is added in a step-wise manner (one after another) up to the last block/model. The final output generates a table where the first model appears in all the subsequent models added and then the second, third, and fourth according to the sequence of addition. This is basically used to demonstrate the improvement in the models as you subsequently add more models. The order of addition of models doesn’t matter as long as the data fit in the model and goodness-of-fit obtained. The levels of statistical significance keep on changing for each variable as you add another model, this has a huge impact on the last variables in the model as a result of interactions among multiple variables. This is shown by changes in the level of significance of the same variable as you move from one model to another. You may also realize that many variables were significant at the crude/unadjusted analysis level but become insignificant in the adjusted analysis level and after you move from one model to another. Some variables were eventually eliminated due to over or under contributing to the models. So, the final/last model is considered the best model after undergoing a rigorous process. This kind of modelling has been regarded in literature and among scholars as the best type of modelling in the regression method.

**Comment 10.** I appreciate the VIF approach used to reducing issues of collinearity. Did you consider whether some variables might be colliders in each model? It would be helpful to have a DAG since there are a lot of variables being tested.

**Response 10.** Colliders in each model was checked using multicollinearity diagnostic test and using a cut-off value of less than 6. This is also supported by literature and scholarly books. The use of 4 models has rigorously catered for colliders.

**Comment 11.** Line 299 states that only one model was discussed. Why?

**Response 11.** Yes, only one model which is the last model (model 4) was discussed. This is because using many models is meant to eliminate colliders, confounders, and limit interactions among different variables. Therefore, the last model is considered as the best model having undergone a rigorous process.

**Comment 12.** Did you perform p-value correction for multiple testing in the unadjusted analysis?

**Response 12.** We did not perform p-value adjustment/correction because our study did not include multiple dependent/outcome variables and simultaneous hypotheses testing. Besides, p-value correction is a conservative test which reduces the chance of making Type I Errors, but increases the chances of making Type II Errors.

**Results**

**Comment 13.** The authors use ‘odds’ and ‘likelihoods’ interchangeably when they should not be.

**Response 13.** We have removed the term likelihoods and remained with strictly odds.

**Comment 14.** Line 324: a higher proportion of what?

**Response 14.** This is corrected to read “a higher proportion of respondents”.

**Comment 15.** There appear to be errors in Table 3. Some of the ORs are not within the confidence intervals [e.g., age 1.61 (0.36-0.98), tobacco smoking 0.38** (1.32-5.33)].

**Response 15.** These were typos but have now been corrected in Table 3 as; age category 0.61 (0.36-0.98) and tobacco smoking 1.38** (1.32-5.33).

**Comment 16.** Setting the reference group for the stigma variable to “never” would increase the interpretability of this variable. Just a thought.

**Response 16.** We did a lot of trial for various reference groups and those we selected were the best in the sub-variables.

**Discussion:**

**Comment 17.** The prevalence of delayed ART initiation is lower in this cohort than is expected from this region according to the studies cited in the introduction. What might be the reasons for this? Is there something about this particular sample/cohort (e.g., better care, better follow-up, etc.) that makes this study population different from the target population?

**Response 17.** Our study showed lowered prevalence of delayed ART initiation due to adherence to recent test and treat policy, follow-up of lost patients, provision of nutritional supplement and basic needs (treated mosquito nets, water treatment products, and container for storage of drinking water) with support from USAID partner (NU-HITES), support from linkage facilitators who performs counselling and referral of persons with HIV in the community. This statement is as well added to the manuscript text.

**Comment 18.** Lines 416-417: ‘….adult HIV patients who were in the age group between 35-44 years were 3.85 times higher to delay ART initiation…’ this statement is confusing. Do you mean the odds of delayed initiation were higher in this age group?

**Response 18.** This has been corrected in the text to read “respondents between the age group 35-44 years had 3.85 higher odds of delayed ART initiation.

**Comment 19.** Why do you suppose the odds of delayed ART initiation were higher among urban vs rural residents in the unadjusted analysis but not in the adjusted analysis?

**Response 19.** We believe in the unadjusted analysis, urban dwellers had higher odds of delayed ART initiation as opposed to the adjusted analysis due to collision or confounders. The explanation could be due to fear of stigma or shame and rejection from friends, workmate, relatives etc. But after adjustment the confounders were eliminated and true picture is as presented.

**Comments 20.** There were a few other findings that need more explanation:
- Why was ‘no education’ protective compared to ‘primary education’?
- Why was stigma protective?
Were these variables truly protective or could the model have been misspecified (e.g., some variables controlled for were actually colliders or other variables were not available)?

**Response 20**

- No education could be protective in the sense that they usually accept messages from the healthcare workers without much resistance than people who have attained from form of education. Majority of them are also poor so, they would want to take advantage of opportunities presented to them.
- As you can see those who never experienced stigma were more protective as compared to those who rarely experienced stigma. The possible explanation of some form of stigma being protective could be as a result of false positive.
- Other possible explanation could also be due to bias, that is, misclassification of variables.

**Comment 21.** The emphasis on cultural acceptance, one of the major findings of this study, is essential. The authors include a detailed discussion on this topic, but the readers are left wondering what can be done about this. Are there any successful approaches available in the literature that can be cited?

**Response 21.** We have provided more discussions about culture in relation to ART services as recommended.

**Response to reviewer 2**

MAJOR COMMENTS

**Comment 1.** The introduction is too long and could be better organized - currently, there are many statements presented that seem quite peripheral to the matter of delayed ART initiation, the focus jumps from Uganda to sub-Saharan Africa to the global pandemic, and the authors do not provide a clear narrative about why their study is important and necessary. I would encourage the authors to reframe the introduction to clearly describe the scope of the problem, what is known about it, and what is unknown (i.e. the gap that their study is addressing) and only retain the necessary parts of the text to communicate this.

**Response 1.** The introduction has been revised and shortened as advised. We have added the justification to the study in the text as “Our study will provide information that can be used to address challenges contributing to delays in ART initiation. It will provide information to health managers, relevant stakeholders, and ministry of health to effectively plan for the implementation of ART services that will contribute to increased access to ART and early ART initiation. The information can also lead to programmatic and policy adjustments related to HIV management among adults”.

**Comment 2.** The sections in the introduction that describe the HIV pandemic and delivery of HIV care in Uganda (much of the text from lines 138-184) can be moved to Methods (Study Context subsection) - would also try to shorten this text substantially.

**Response 2.** This has been corrected as recommended in the methods section under study context.

**Comments 3.** It is not entirely clear to me who was included in the study population, described in their sampling strategy in the Study Design and Sampling Procedure section. The authors present an N of 831 as the “population size” - unclear what, was this all newly diagnosed PLWH receiving care in the study centers? They also note on lines 213-215 that “all individuals tested…were included in the study.” Can they provide a clearer description of the OVERALL sample from which the study population was derived?

**Response 3.** 831 is the total number of clients enrolled on ART in the district. The phrase “all individuals tested….were included in the study” was an oversight but we meant they were included in the sample. This has been corrected in the text.

**Comment 4.** Did patients need to have started ART in order to be included in the study? From the text in lines 270-273 it seems that patients who never started ART were included in the “Delayed ART initiation” group. I would recommend that patients that never initiated ART be their own group or not included in the study.

**Response 4.** According to our definition of delayed ART initiation i.e. initiation of ART after 30 days of confirmed HIV testing. Our study included those who were initiated on ART after 30 days of confirmed HIV testing.

**Comments 5.** The authors could provide a little more clarity with respect to some of the covariates included in the study: 1) what is “frustration with HIV results?;” 2) why include both time to reach health facility (as an individual characteristic) and distance to health facility (as a community characteristic)? - These seem redundant; 3) why is experience of stigma a community characteristic rather than an individual one?

**Response 5.**

- Frustration with HIV results – in this context means the feeling of being annoyed or less confident because of discovering one is with HIV/living with HIV. This was well explained during data collection.
- In table 4, time to reach health facility is an individual character because time management can be independently controlled by an individual. Distance to the health facility which is related to the located of the health facility. In Uganda the decision to locate a health facility is decided upon by the community leaders.
- Stigma can be both an individual and community characteristic. But the one mostly affecting health service delivery in Uganda is community stigma – it has a huge impact on both the patient and healthcare service delivery.

**Comment 6.** The authors provide criteria for inclusion of variables in the multivariable analysis in the methods (288-290), but it seems as if all of the variables were included in both bivariate and multivariate models, even though some were clearly not significant in bivariate analyses and seem peripherally related to ART initiation. Can the authors either justify this or present a more streamlined multivariate analysis?

**Response 6.** We had so many variables in our questionnaire as can be seen in the attachment. However, some of them were eliminated at bivariable level using Chi-square test and a cut-off p-value of less than 0.25. This was the basis of our variable selection for logistic regression at both bivariable (unadjusted) and multivariable (adjusted) levels. Some variables were further eliminated due to the multicollinearity effect as specified in the text.

**Comment 7.** Are the results presented in Table 4 accurate? For age >35 and no education, higher proportions of patients had delayed ART starts, but the odds of delayed ART were significantly lower. Similarly, for patients that never experienced stigma, a lower proportion of patients had delayed ART start, but odds of delayed ART were higher. This is problematic.

**Response 7.** We have made corrections to all the errors identified as specified in the manuscript text.

**Comment 8.** The Discussion is too long, and the points that the authors are making are not always well-contextualized. Some of the literature that they choose for comparison is old and the comparisons are thus not entirely valid. The interpretations about why their results differ from others sometimes feels speculative (for example, lines 448-451). I would recommend that the authors first check their models to ensure accuracy (see prior point), the choose 3 of the most important findings, and then carefully review the literature not only for prior findings but for potential reasons why there may be differences or similarities.

**Response 8.** The Discussion section in the manuscript has been improved as recommended.

**Comment 9.** The authors note that the observed rate of delayed ART is much lower than previous studies, however, they fail to note that 3 of the 4 studies they cite were from the pre-Treat All era. Currently and recently (i.e. during the study period), it is commonplace for patients to initiate ART on the day of diagnosis or very shortly thereafter. Therefore, they should make comparisons to studies conducted within the last 4-5 years, after global Treat All implementation.

**Response 9.** We have added more recent articles and dropped older ones as recommended.

MINOR COMMENTS
**Comment 10.** No need to spell out HIV or AIDS, these are universally understood acronyms.

**Response 10**. We have taken care of the recommended as shown in the text.

**Comment 11.** What is HMIS (line 213)?

**Response 11.** HMIS means Health Management Information System. This has been included in the text.

**Comment 12.** Lines 282-288 describing logistic regression are not necessary.

Instead of writing “less likely odds of…” I would suggest “lower odds of….”

**Response 12.** We have corrected in the text as recommended.

**Comment 13.** Lines 411-414 in the Discussion are very unclear.

**Response 13.** We have improved on our explanations as indicated in the text.
