# Supplementary material for: Prevalence and factors associated with delayed antiretroviral therapy initiation among adults with HIV in Alebtong district, Northern Uganda: A facility-based study
Source: PLOS Glob Public Health. 2022 Aug 8;2(8):e0000691. doi: 10.1371/journal.pgph.0000691 (PMC10021445; doi:10.1371/journal.pgph.0000691)
Supplement: Attachment — Submitted filename: Response to Reviewers Comments.docx [file pgph.0000691.s006.docx]

**Response to Reviewers**

ABSTRACT

-No need to spell out HIV and AIDS acronyms, these are universally recognized

**Response.** This is corrected as shown in the manuscript.

-Can abbreviate people living with HIV as PLWH or PLHIV

**Response.** We have abbreviated people living with HIV as “PLWH”

-Not clear what they mean by “adherence to HIV clinic health facility 78 appointments, (aOR = 0.06, 95% CI: 0.02-0.15); and linkage to care same day tested (aOR = 79 0.21, 95% CI: 0.08-0.55)” - can the language be clarified?

**Response.** We meant “adherence to HIV clinic appointments” and “linkage to the HIV clinic the same day HIV test was conducted”. This has been revised.

INTRO

-The authors were responsive in cutting extraneous text in the introduction.

**Response.** Thank you

-I would recommend leading the introduction with the second paragraph (Lines 86-90; “Globally, an estimated 36.7 million…”) and then following with the text about ART

**Response.** We have deleted the first paragraph and started with the second paragraph as suggested.

-The authors present a number of factors that are already associated with delayed ART initiation (lines 75-85) - is there a reason to believe that these would be different in Alebtong district? In other words, is there a research gap if these factors are already known? This would add to the motivation for this study, in addition to the text the authors already added in line 111-117.

**Response.** Lines 75-85 have already been deleted from the manuscript as suggested and we have started our intro with the second paragraph as indicated in the manuscript. The factors are not known yet but we had demonstrated possible factors in line with literature from other studies. We have also added more justification for the study as shown in lines 101-103 in the manuscript.

METHODS

-I remain somewhat confused by the description of sampling and data collection, as the amount of detail is overwhelming at times and it is difficult to follow. Perhaps the authors can include a flowchart as a supplementary figure outlining the steps undertaken (i.e. sample size determination, sampling, final sample size, and steps in data collection)?

**Response.** The seven health facilities offering ART services were identified using a simple random sampling method and samples from each health facility were selected based on probability proportional-to-size sampling. This is because the health facilities had unequal samples; some had small number of PLWH on ART and others had bigger numbers that were not the same. A flowchart of sampling procedures is provided. Data collection procedures are provided in lines 188-202.

-The authors do not clearly define what the inclusion and exclusion criteria were for the study

**Response.** The inclusion and exclusion criteria have been added in lines 152-156.

-It remains unclear if the data on dates was extracted from medical records or some other source?

**Response.** The data on dates was extracted from the medical records “ART registers”

-In Tables 3 and 4, in referring to religion and employment, the authors need to use other symbols for the footnotes besides asterisks (*, **) as their repeated use is confusing.

**Response.** We have corrected as asterisks (others^a^ = employment and others^b^ = religion) as shown in Table 3 and 4.

DISCUSSIONS

-The authors continue to make comparisons between their results with studies that are not valid comparators. They compare the prevalence of delayed ART of 18% in their study to that in Ethiopia in the era prior to Treat All, when guidelines did not emphasize rapid initiation (ref 39), or to a study of inpatients in Uganda (ref 40). Any comparison with respect to the proportion of patients initiating late should come from studies examining general populations in the Treat All era.

**Response.** We have removed references 39 & 40 since they are not in line with the test and treat guidelines.

-It is speculative to attribute the low rate of delayed ART to interventions such as nutritional supplements, treated mosquito nets, and other factors that were not measured in the study.

**Response.** We have edited our explanations and also removed texts that appear speculative i.e. interventions such as nutritional supplements, treated mosquito nets. Check lines 346-348.

-Similarly, the authors interpretation of their results with respect to age and adherence to appointments as predictors of delayed ART initiation (375-386, and 387-400) feels very speculative. Would suggest editing this carefully.

**Response.** We have carefully edited the explanations as seen in lines 353-362.
